# Therapies for multiple sclerosis targeting B cells

**DOI:** 10.3325/cmj.2019.60.87

**Published:** 2019-04

**Authors:** Ron Milo

**Affiliations:** 1 Department of Neurology, Barzilai Medical Center, Ashkelon, Israel; 2 Faculty of Health Sciences, Ben-Gurion University of the Negev, Beersheba, Israel

## Abstract

Increasing evidence suggests that B cells contribute both to the regulation of normal autoimmunity and to the pathogenesis of immune mediated diseases, including multiple sclerosis (MS). B cells in MS are skewed toward a pro-inflammatory profile, and contribute to MS pathogenesis by antibody production, antigen presentation, T cells stimulation and activation, driving autoproliferation of brain-homing autoreactive CD4+ T cells, production of pro-inflammatory cytokines, and formation of ectopic meningeal germinal centers that drive cortical pathology and contribute to neurological disability. The recent interest in the key role of B cells in MS has been evoked by the profound anti-inflammatory effects of rituximab, a chimeric monoclonal antibody (mAb) targeting the B cell surface marker CD20, observed in relapsing-remitting MS. This has been reaffirmed by clinical trials with less immunogenic and more potent B cell-depleting mAbs targeting CD20 – ocrelizumab, ofatumumab and ublituximab. Ocrelizumab is also the first disease-modifying drug that has shown efficacy in primary-progressive MS, and is currently approved for both indications. Another promising approach is the inhibition of Bruton's tyrosine kinase, a key enzyme that mediates B cell activation and survival, by agents such as evobrutinib. On the other hand, targeting B cell cytokines with the fusion protein atacicept increased MS activity, highlighting the complex and not fully understood role of B cells and humoral immunity in MS. Finally, all other approved therapies for MS, some of which have been designed to target T cells, have some effects on the frequency, phenotype, or homing of B cells, which may contribute to their therapeutic activity.

Traditionally, multiple sclerosis (MS) has been considered an autoimmune disease of the central nervous system (CNS) mediated by CD4+ T cells reactive to myelin antigens (1). This theory is supported by data from animal models (2), the association of MS with certain human leukocyte antigen (HLA) alleles that are critical for T cell activation (3), genome-wide association studies (4), and immune alterations in individuals with MS (5). The role of B cells in MS has long been ignored, despite evidence for the presence of elevated antibodies in the cerebrospinal fluid (CSF) of MS patients (6), the discovery of oligoclonal bands (OCBs) in the CSF, which indicate local production of immunoglobulins by oligoclonal B cells in the CNS (7), and the presence of B cells and plasma cells expressing hypermutated immunoglobulins in MS lesions (8). The surprising anti-inflammatory effect exerted by rituximab, a chimeric monoclonal antibody (mAb) targeting CD20 (a B cell marker) in patients with relapsing-remitting MS (RRMS) shed light on the key contribution of B cells to neuroinflammation (9). Recent advances in flow cytometry and DNA-sequencing methods have made it possible to analyze B cells in the CNS and to unveil their central role in the MS pathogenesis.

## ROLE OF B CELLS IN MS

T cells are traditionally viewed as playing a key role in the immune pathogenesis of MS, where imbalance between CNS-reactive effector T cells of the helper-1 (Th1) and Th17 type and regulatory T cells (Treg) underlies autoimmunity directed at the CNS (10). According to this view, myeloid cells, either pro-inflammatory M1 macrophages (secreting interleukin [IL]-12, IL-23, IL-6, and IL-1β) or anti-inflammatory M2 macrophages (secreting IL-10), shape T cell response, while their own responses may be shaped by differentiated T cells. In this scenario, B cells were considered to be a relatively homogenous and passive population, awaiting the help of T cells to differentiate into plasmablasts and plasma cells that contribute to MS pathophysiology by producing CNS-autoreactive antibodies. Recent research, however, has led to an emerging view of a broader and more central role of B cells in MS, which is mainly antibody-independent. B cells can have several phenotypes according to their cytokine profile and manifest as either pro-inflammatory effector B cells (secreting TNF-α, lymphotoxin-β [LT-β], interferon γ [IFN-γ], IL-6, IL-15, and granulocyte macrophage colony stimulating factor [GM-CSF]) or anti-inflammatory regulatory B cells (Breg, secreting IL-10, transforming growth factor-β [TGF-β], and IL-35), which either activate or down-regulate the responses of both T-cells and myeloid cells. Thus, complex bidirectional interactions among functionally distinct populations of T cells, B cells, and myeloid cells, some of which may be over-active or hypo-functional in MS, underlie and shape CNS-directed autoimmunity (11).

Peripheral mature B cells can cross the blood-brain-barrier (BBB) into the CNS via parenchymal vessels into the perivascular space and via post-capillary venules into the subarachnoid and Virchow-Robin spaces. They can also cross the blood-cerebrospinal fluid (CSF) barrier via the choroid plexus into the CSF, and via the blood-leptomeningeal interphase (12). In the CNS, a restricted number of expanded clones of B cells and plasma cells produce immunoglobulins and form oligoclonal bands (OCBs) observed in most MS patients (13). These clones tend to persist within the CNS and can be shared among different CNS compartments and the periphery, suggesting bidirectional trafficking of distinct B cell clones between the CNS and the periphery (11). Thus, B cells can dynamically traffic into and out of the CNS via the recently-discovered functional lymphatic vessels that are lining the dural sinuses, can potentially carry, process, and present CNS antigens in the deep cervical lymph nodes, make their way back into the CNS via the thoracic duct, systemic circulation, and the various brain barriers, infiltrate the brain parenchyma, populate ectopic lymphoid follicles, and trigger another bout of CNS-targeted inflammation (12).

B cells can contribute to MS pathogenesis by several ways, including antibody production, antigen presentation and activation of T cells, cytokine production, and formation of ectopic germinal centers.

### Antibody production

OCBs of the IgG type are present in most patients with MS, and OCBs of the IgM type are present in 30%-40% of patients. These OCBs are made up by plasma cells generated from a restricted numbers of B cell clones that persist within the CNS of the same individual and are shared by different CNS compartments and the periphery, but differ among individual patients (11,13). The antibodies that make up these OCBs primarily recognize ubiquitous intracellular proteins but not specific antigens that are shared across MS patients, suggesting a humoral response to debris from dead-cells rather than a primary pathogenic response (14). Antibodies to myelin antigens or to the potassium channel KIR4.1 found in MS patients do not seem to have any pathogenic role (15,16). Moreover, the rapid decrease in clinical and MRI disease activity after B cell depletion (9) is unlikely to result from the removal of any pathogenic antibodies, which have relatively long half-life. Taken together, these data suggest no major pathogenic role for antibodies produced by B cells and plasma cells in MS. On the other hand, anti-myelin/oligodendrocyte glycoprotein antibodies have been shown to contribute to demyelination in the experimental allergic encephalomyelitis (EAE) model (17), and demyelinating MS lesions contain immunoglobulins and activated complement, which may suggest antibody-mediated damage at least in some patients (18).

### Antigen presentation

B cells express high levels of major histocompatibility complex molecules on their surface, which present short linear epitopes to T cells. They also express membrane-bound antigen-specific immunoglobulins, which correspond to the soluble immunoglobulins they secrete after developing into plasma cells or plasmablasts. These B cell receptors (BCRs) can recognize and bind three-dimensional conformational epitopes. Cognate antigen presentation by resting B cells promotes T-cell tolerance, while B cells activated by antigen and T cells become antigen presenting cells (APC) capable of promoting immune responses (19). Thus, B cells are highly efficient APC, particularly when they recognize the same antigen as T cells, and appear to be the main source of APCs when antigen levels are low.

Another mechanism for B cells to create and maintain pathogenic T cell repertoire is autoproliferation (which refers to the activation and growth of myelin-specific T cells by APC in the absence of exogenous nominal antigen), which was found to be increased in MS patients. Autoproliferation in MS patients carrying the HLA-DR15 haplotype was found to be driven by memory B cells in a HLA-DR-dependent manner and reduced by B cell depletion with anti-CD20 (20). Furthermore, the autoproliferating T cells were enriched for brain-homing, probably pathogenic T cells, and a target autoantigen, RASGRP2, was found to be expressed in both the brain and B cells (20).

### Cytokine production

Patients with MS show aberrant B cell cytokine response to stimuli and produce abnormally high amounts of pro-inflammatory cytokines (eg, IFN-γ, TNF-α, LT-α, IL-6, and GM-CSF), which may activate T cells and myeloid cells and contribute to the disease process (21). TNF-α secreted by B cells can also stimulate the secretion of the cytokine B cell activating factor (BAFF) by astrocytes, the expression of which is increased in MS lesions, thus enhancing B cell dependent autoimmunity. B cell depletion with anti CD20 mAbs abrogates B -cell inflammatory responses and decreases inflammatory responses of both T cells and myeloid cells, highlighting the close interactions between the three cell types and the contribution of cytokines secreted by pro-inflammatory B cells to MS pathogenesis, independent of their antibody-production function (11). B cells can also down-regulate immune responses and limit CNS inflammation through the secretion of anti-inflammatory cytokines (eg, IL-10, TGF-β, and IL-35) by Breg, which were found to be defective in MS (22). Overall, B cells in MS are skewed toward a pro-inflammatory cytokine profile, which can drive T cells and myeloid cells and enhance pathogenic immune responses.

### Formation of ectopic germinal centers

B cells that have been attracted to the brain of MS patients under stimuli such as CXCl13, with the appropriate help from T cells, can proliferate, aggregate, and generate meningeal inflammation and eventually ectopic immunocompetent germinal center-like structures, called also tertiary lymphoid organs, which are associated with more severe cortical pathology and more aggressive disease course (23). These B cell-rich ectopic lymphoid structures, which were described in secondary-progressive (SP) MS (23), RRMS (24), and active primary-progressive MS (PPMS) (25), can serve as a reservoir of memory-B cells and autoreactive plasmablasts and plasma cells, perpetuating autoimmune disease. In addition, they can secrete soluble factors that were shown to be cytotoxic to both oligodendrocytes (26) and neurons (27).

## MS THERAPIES TARGETING B CELLS

The most effective and studied therapies targeting B cells include mAbs that deplete B cells through mechanisms of antibody-dependent cellular cytotoxicity (ADCC), complement-dependent cytotoxicity (CDC), and antibody-triggered apoptosis. Other strategies include the targeting of B cell cytokines or their receptors and inhibition of Bruton's tyrosine kinase (Table 1). In addition, all other MS therapies have been found to exert suppressive or immunomodulatory effects on B cells (Table 2).

**Table 1:** Main clinical trials with B cell-directed therapies*

Trial	Drug name, mode of action	Route	Phase	Type of MS	No. of patients	Design	Main clinical outcomes	Main MRI outcomes	Main adverse events
HERMES (36)	rituximab, anti-CD20 chimeric IgG mAb	IV	2	RR	104	DB, PC, 48- week	↓ARR (week 24, 14.5% vs 34.3%, *P* = 0.02; week 48, 20.3% vs 40.0%, *P* = 0.04)	↓Total Gd + lesions (*P* < 0.001) ↓Total new gadolinium-enhancing lesions (*P* < 0.001) ↓Lesion volume on T2WI	IAR – 40% vs 20% Infections – 70% in both groups
OLYMPUS (37)	rituximab	IV	2/3	PP	439	DB, PC, 96- week	No difference in 12 week CDP; Delayed time to CDP in patients aged <51, those with Gd + lesions at baseline, or both	↓Increase in T2 lesion burden No difference in brain volume change	IAR (mild to moderate) Infections
OPERA I+II (40)	ocrelizumab, anti-CD20 humanized IgG1 mAb	IV	3	RR	1656	DB, DD, comparator-controlled (SC IFNβ-1a), 96-week	↓ARR by 46% and 47% (*P* < 0.001) ↓40% in 12 week CDP (*P* < 0.001) and 24 week CDP (*P* = 0.003) NEDA – 48% for OCR-treated patients over 2 years; 72% (weeks 24-96)	↓Mean number of Gd + lesions (94% and 95%). ↓Total mean number of new or newly enlarging T2 lesions (77.3% and 82.6%)	IAR – 34% vs 9.7 (mild to moderate); Infections – 56.8% (OCR), 53.4% (IFNβ-1a); Serious infections – 2.9% (IFNβ-1a), 1.3% (OCR); Neoplasms – 0.5% (OCR) 0.2% (IFNβ-1a)
ORATORIO (44)	ocrelizumab	IV	3	PP	732	DB, PC (2:1), 120- week	↓12 week CDP (32.9% vs 39.3%, *P* = 0.03); ↓24 week CDP (29.6% vs 35.7%, *P* = 0.04); NEPAD – 3 fold OCR vs placebo	↓Total volume of hyperintense T2 lesions (-3.4 vs 7.4, *P* < 0.001); ↓Adjusted mean number of new or enlarging hyperintense T2 lesions (0.31 vs 3.88, *P* < 0.001); ↓Mean percentage change in brain volume (-0.90 vs -1.09, *P* = 0.002)	IAR – 40% (mild to moderate); Infections – 71.4% (OCR), 69.9% (placebo). Serious infections – 6.2% (OCR), 5.9% (placebo); Neoplasms – 2.3% (OCR), 0.8% (placebo).
MIRROR (49)	ofatumumab, anti-CD20 fully human IgG1 mAb	SC	2	RR	232	DB, PC, 48-week	↓ARR No difference in disability outcomes	↓Mean rate of cumulative new Gd + lesions (65% for all doses between weeks 0-12, *P* < 0.01)	Injection-related reactions – 97% (mild to moderate)
(51)	ublituximab, anti-CD20 glycoengineered chimeric IgG1mAb	IV	2	RR	48	DB, PC, 48-week	ARR – 0.07; Relapse-free – 93% CDP at week 24 – 7% CDI at week 24 – 17% NEDA – 74%	↓100% of Gd + lesions ↓10% in mean T2 lesion volume	IAR (mild to moderate)
(68)	inebilizumab (MEDI-551), anti-CD19 glycoengineered humanized IgG1κ mAb	IV or SC	1	RR	28	PC, 24-week, dose-escalation		↓New Gd + and new or newly enlarging T2 MRI lesions	IAR – 40% of patients on inebilizumab or placebo; Injection site reactions – 17%; Infections
ATAMS (58)	atacicept (TACI-Ig), fusion protein (TACI receptor and Fc domain of human IgG1)	SC	2	RR	255	DB, PC, 36-week	↑ARR in all 3 atacicept groups; Trial prematurely terminated	Similar mean numbers of Gd+ T1 lesions per scan in all groups	Injection site reactions More SAE in the atacicept groups
ATON (59)	atacicept	SC	2	ON	34	DB, PC, 36-week	More atacicept-treated patients converted to clinically-definite RRMS (35.2%) than placebo-treated patients (17.6%) despite having less retinal axonal loss	NA	Injection site reactions No SAE
(64)	evobrutinib, BTK inhibitor	oral	2	RR	267	DB, PC, 36-week	A trend toward a reduction in ARR	↓T1 Gd + lesions	**↑**Liver enzymes (asymptomatic)

*ADCC – antibody-dependent cellular cytotoxicity; ARR – annualized relapse rate; BCR – B cell receptor; BTK – Bruton's tyrosine kinase; CDC – complement-dependent cytotoxicity; CDI – confirmed disability improvement; CDP – confirmed disability progression; DB – double-blind; DD – double dummy; Gd – gadolinium; IAR – infusion-associated reactions; IFN – interferon; IV – intravenous; mAb – monoclonal antibody; MS – multiple sclerosis; NA – not available; NEDA – no evidence of disease activity; NEPAD – no evidence of progression or active disease; OCR – ocrelizumab; ON – optic neuritis; PC – placebo-controlled; PP – primary-progressive; RR – relapsing-remitting; SAE – serious adverse events; SC – subcutaneous; T2WI – T2 weighted images; TACI – transmembrane activator and calcium modulator and cyclophilin ligand interactor.

**Table 2:** Effects of other approved disease modifying therapies on B cells*

Drug name	Target/mode of action	Effect on B cells (11,32,57,65-67)
**Interferon-β**	Immunomodulatory effects on various immune cells and molecules	↓mB cells, nB cells expressing CD86 and CCR5 ↑IL-10 producing Breg, TGF-β
**Glatiramer acetate (GA)**	Immunomodulation: generation of GA-specific Th2 cells, inhibition of myelin-specific Th1 cells, modulation of myeloid cells	↓mB cells, CXCR5and ICAM-3 in B cells; ↓IL-6, LT-α, and TNF-α ↑IL-10 producing Breg
**Mitoxantrone**	Topoisomerase II inhibitor, suppression of immune cell proliferation	↓B cells, ↓mB cells, ↓TNF-α and LT-α, ↑IL-10
**Natalizumab**	Anti-VLA-4, prevention of leukocyte trans-migration into the CNS	Blood: ↓nB cells, ↑Breg cells, mB cells CSF: ↓B cells, immunoglobulins, OCBs
**Fingolimod**	S1P-R modulator, prevention of lymphocyte egress from lymph nodes	Blood: ↓nB cells, mB cells CSF: Minor decrease only in the number of B cells, ↑Breg Abrogation of B cell aggregate formation in the CNS (EAE)
**Teriflunomide**	Inhibition of DHODH and *de-novo* pyrimidine synthesis	↓B cells proliferation and activation, ↓B cells in blood, ↓IL-6, IL-8
**Dimethyl fumarate**	Activation of NRF2 pathway, inhibition of NFκB pathway	↓mB cells, ↓GM-CSF, IL-6, TNFα, ↑Breg
**Cladribine**	Impairment of DNA synthesis, lymphocyte apoptosis.	Depletion phase: ↓B cells in blood Reconstitution phase: ↓mB cells
**Alemtuzumab**	Anti-CD52, lymphocyte depletion	Depletion phase: ↓B cells Reconstitution phase: ↑B cells (tB cells, nB cells, Breg)

*Breg – regulatory B cells; CCR5 – C-C chemokine receptor 5; CNS – central nervous system; CSF – cerebrospinal fluid; CXCR – CXC chemokine receptor; DHODH – dihydroorotate dehydrogenase; EAE – experimental autoimmune encephalomyelitis; GM-CSF – granulocyte-macrophage colony-stimulating factor; ICAM-3 – intracellular adhesion molecule-3; IL – interleukin; mB cells – memory-B cells; nB cells – naive B cells; NRF2 – nuclear factor erythroid 2-related factor 2; NF-κB – nuclear factor kappa light chain enhancer of activated B cells; OCB – oligoclonal bands; S1P-R – sphingosine-1-phosphate receptor; tB cells – transitional B cells; TGF-β – transforming growth factor beta; TNF-α –tumor necrosis factor alpha; VLA-4 – very late antigen 4.

### Anti CD20 mAbs

CD20 is a transmembrane ion channel protein expressed on the surface of pre-, immature-, mature-, and memory-B cells, and to a lesser extent – on early plasmablasts, but not on stem cells, pro-B cells, late plasmablasts, or plasma cells (28). Anti-CD20 therapies rapidly and almost completely deplete circulating CD20+ B cells, but limitedly penetrate lymphoid organs. B cell reconstitution from stem cells and pro-B cells in the bone marrow, and preexisting humoral immunity and antibody production from late plasmablasts and plasma cells, are largely preserved. Although anti-CD20 mAbs almost do not cross the BBB, they eliminate B cells in the CSF without a detectable effect on the IgG index or oligoclonal bands (29). About 5%-7% of the total mature circulating T cells also express CD20 and can be depleted by anti-CD20 mAbs but do not appear to have a particular role in MS disease activity. Following depletion, mainly naive and immature B cells are reconstituted, while memory-B cells are suppressed and remain low for at least 1-2 years, pro-inflammatory cytokines (GM-CSF, TNF-α, LT- α) decrease, and B regulatory cells producing anti-inflammatory cytokines increase (30-32). Anti-CD20 treatment also alters T cell function and markedly reduces the proliferation and pro-inflammatory cytokine production of CD4+ and CD8+ T cells (33), while increasing regulatory T cells (34). These quantitative and qualitative changes in both cellular and humoral arms of the adaptive immune system clearly form the basis for the therapeutic efficacy of anti-CD20 mAbs in MS.

Four anti-CD20 mAbs have been studied in MS so far: rituximab, ocrelizumab, ofatumumab, and ublituximab, which differ from each other not only by their structure and immunogenicity (chimeric, humanized, fully human, or glycoengineered, respectively), but also by the relative degree of ADCC and CDC they exert and the CD20 epitope they recognize (Table 1).

*Rituximab.* Rituximab is a chimeric IgG1 mAb, depleting B cells primarily through CDC. It was first studied in a small open-label, phase-I, multicenter clinical trial of 26 RRMS patients treated with two courses of rituximab 24 weeks apart. After 18 months, relapses were reduced by more than 80%, and fewer new MRI gadolinium-enhancing (Gd+) or T2 lesions were observed (35).

In the phase-II HERMES trial, 104 patients with RRMS were randomized (2:1) to receive either a single course of intravenous (IV) rituximab or placebo on days 1 and 15. Rituximab reduced newly MRI Gd + lesions by more than 90%, which was sustained at 48 weeks, and reduced relapse rate by more than 50% (36).

In the OLYMPUS phase II/III study, 439 patients with PPMS were randomized 2:1 to receive either 4 courses of two 1000-mg intravenous rituximab or placebo infusions every 24 weeks. Although the primary endpoint, time to confirmed disability progression (CDP) sustained for 12 weeks was not met, patients treated with rituximab had less increase in T2 volume load on MRI (*P* < 0.001). Subgroup analysis showed that time to 12 week CDP was delayed in patients aged <51 and/or patients with Gd + lesions in the rituximab group compared with placebo, suggesting a beneficial effect of B cell depletion in younger PPMS patients with inflammatory activity (37).

The lack of efficacy of rituximab in PPMS may be attributed to the very low concentrations in the CSF achieved after IV administration, insufficient to affect the compartmentalized CNS inflammation, which arguably drives progressive MS. Thus, the effect of double-blind combination of rituximab by IV and intra-thecal (IT) injection vs placebo was tested in the RIVITALISE study (38). Although IT rituximab nearly completely depleted B cells in the CSF, this effect lasted only 3 months, B cells in CNS tissue were inadequately depleted, T cells were not depleted, and neurofilament light chain (a marker for axonal damage) did not change. Lower CSF rituximab concentrations with insufficient saturation of CD20, partial ADCC killing, lack of lytic complement with poor CDC, and paucity of cytotoxic CD56(dim) natural-killer (NK) cells contributed to decreased efficacy of rituximab in the CNS (38). This trial was ultimately halted but provided more evidence for the difficulty of targeting the inflammatory process in the CNS and meninges.

The development of rituximab for MS has never been completed for a variety of reasons, and attention has been shifted to less immunogenic and potentially more potent, humanized and fully human anti CD20 mAbs. Nevertheless, rituximab is still used off-label (39).

*Ocrelizumab.* Ocrelizumab is a humanized IgG1 mAb that depletes B cells primarily through enhanced ADCC activity due to the higher affinity of its humanized Fc region for the FcγRIIIa receptors present on NK cells and macrophages. Ocrelizumab was tested in two identical phase III clinical trials (OPERA I and II) using a dosage of 600 mg (300 mg given twice over 2 weeks with subsequent re-dosing given as a single 600 mg dose every 6 months) (40). It reduced annualized relapse rate (ARR) by 46% and 47%, respectively, compared with IFN-β-1a 44 μg administered three times weekly. Pooled analyses showed a reduction of 40% in the percentage of patients with CDP at 12 weeks and 24 weeks. Moreover, more patients in the ocrelizumab group showed confirmed disability improvement (CDI) than in the IFNβ-1a group. Ocrelizumab reduced the total mean number of Gd + lesions by 94%-95%, the number of new or enlarging T2 lesions by 77%-83%, and significantly reduced the rate of brain volume loss. No evidence of disease activity (NEDA, defined as no clinical relapse, no 12-week confirmed disability progression, and no radiological activity) was achieved by 48% of ocrelizumab-treated patients over 2 years (40) and by 72% for weeks 24-96 (41).

In the open-label extension study of the OPERA trials, the beneficial effects of ocrelizumab on all outcome measures were sustained in patients continuing ocrelizumab, and patients who switched from IFNβ-1a to ocrelizumab had rapid and robust reductions in ARR and MRI disease activity. Fewer patients who initiated ocrelizumab treatment earlier than those who switched to ocrelizumab later had disease progression, highlighting the importance of early effective treatment in reducing disability progression (42,43).

In contrast to rituximab, the ORATORIO trial showed a significant reduction in disability progression in patients with PPMS treated with ocrelizumab (44). In this trial, 732 patients with PPMS were randomized 2:1 to receive either IV ocrelizumab 300 mg given 2 weeks apart or IV placebo every 24 weeks for at least 120 weeks. There were 24%, 25%, and 29.3% reductions in the 12 week CDP, 24 week CDP, and worsening in the timed 25-ft walk, respectively, in ocrelizumab-treated patients. Ocrelizumab also decreased the number and volume of MRI lesions and brain volume loss (44).

Although the effect of ocrelizumab in PPMS was only modest, it was sustained, and patients in the extension study initiating ocrelizumab between 3-5 years earlier had significant and sustained reductions in disability progression compared with patients switching from placebo after 144-240 weeks (45). In *post-hoc* analyses, ocrelizumab increased 3-fold the proportion of PPMS patients achieving a novel combined measure of NEPAD (no evidence of progression or active disease), defined by the absence of both progression and inflammatory disease activity, which may represent a measure of disease control that is sensitive and meaningful in patients with PPMS (46). Ocrelizumab also reduced the progression of upper limb disability in more disabled or older patients, a finding that set the stage for a larger ORATORIO-HAND study, which is intended to further investigate the efficacy of ocrelizumab in improving upper limb function (47).

*Ofatumumab.* Ofatumumab is a fully human IgG1 mAb that binds a completely distinct epitope from that of rituximab or ocrelizumab. It dissociates more slowly from the CD20 antigen, and exhibits pronounced CDC activity, relatively decreased ADCC, and a low immunogenic risk profile. An initial small trial in RRMS showed profound B cell depletion and suppression of inflammatory disease activity by all three doses of ofatumumab administrated intravenously (48). After the development of a subcutaneous formulation of ofatumumab, the MIRROR trial was conducted. In this phase-II trial, 232 patients with RRMS were randomized to subcutaneous ofatumumab 3, 30, or 60 mg every 12 weeks, ofatumumab 60 mg every 4 weeks for 24 weeks, or placebo followed by ofatumumab 3 mg at week 12 (49). New Gd + lesions were reduced by 65% at all doses, and dose-dependent B cell depletion and reconstitution were observed, indicating that complete depletion was not necessary for a robust treatment effect. The subcutaneous administration of ofatumumab may have the advantages of more convenient self-administration of the treatment at home, but compliance may not be well-controlled and monitored by the treating physician as with IV administration. Two identical phase-III clinical trials in RRMS (ASCLEPIOS I+II) are currently in progress.

*Ublituximab*. Ublituximab is a novel chimeric glycoengineered IgG1 that binds a unique epitope on CD20 and demonstrates increased binding capacity to CD20. It also demonstrates enhanced target cell killing due to defucosylation of its Fc region, which increases the affinity for FcγRIIIa, resulting in more efficient immune effector cell engagement and enhanced target cell killing through ADCC (50). Ublituximab was recently tested in a phase-II, 48-week, placebo-controlled study, which was designed to assess its optimal dose and infusion time in 48 patients with relapsing forms of MS (51). Median B cell depletion was >99% in all patient cohorts. Gd + lesions were reduced to zero; mean T2 lesion volume decreased by 7.3% and 10.6% at week 24 and 48, respectively; 7% of participants had 24-week CDP, 17% met the criteria for 24 week CDI, and 74% met the criteria for NEDA. A rapid one-hour infusion time of 450 mg ublituximab was well tolerated and produced high levels of B cell depletion (51). This regimen is now being studied in two identical phase-III ULTIMATE trials.

### Adverse effects of B cell depletion

The most common side effects of infused anti-CD20 mAbs are infusion reactions, mostly of mild to moderate severity (36,37,40,44,51). These reactions, the result of B cell lysis with massive cytokine release, present most often with the initial dose, tend to decrease with subsequent doses, and can be mitigated by pretreatment with steroids, antihistamines, and acetaminophen.

The risk of infection is an important consideration with profound B cell depletion. Infections were reported in 57%-60% of ocrelizumab-treated RRMS patients compared with 53%-54% of IFNβ-1a patients, with no difference in serious infections (40), and in 71% of ocrelizumab-treated PPMS patients compared with 70% in the placebo group (44). Patients are recommended to be pre-screened for tuberculosis, hepatitis B and C, and HIV, which are of particular concern with B cell depletion, and should not receive live vaccines during B cell depletion therapies.

Several cases of progressive multifocal leukoencephalopathy (PML) have been described in MS patients treated with ocrelizumab, all carried-over from previous natalizumab or fingolimod treatment (52). In rheumatoid arthritis, where rituximab is administered as an add-on therapy, generally with steroids and other immunosuppressants, the risk of PML is estimated at 1:25 000 (53). However, no PML has been described in rituximab-treated MS patients (39) or ocrelizumab-treated patients in clinical trials (40,44).

In the ocrelizumab phase-III trials, an imbalance in the incidence of malignancies was observed. Neoplasms occurred in 0.5% and 2.3% of ocrelizumab-treated patients, compared with 0.2% and 0.8% of IFN and placebo patients, in the OPERA (40) and ORATORIO (44) trials, respectively, with the most frequent malignancy being breast cancer. On the other hand, the trend of increased malignancy, including breast cancer, has fallen in open-label extension studies, and ocrelizumab-treated patients showed no higher incidence of malignancies compared with large cohorts and registries of MS patients (54). Further long-term studies are needed to determine the risk of malignancy with B cell depletion.

### Cytokine antagonists

The main regulatory cytokines of B cell survival, maturation, and activation are BAFF and APRIL (A ProlifeRation Inducing Ligand) (55), which are elevated in patients with MS (56). These molecules are capable of binding 3 separate receptors on B cells with different affinities: BAFF-R, transmembrane activator and calcium modulator and cyclophilin ligand interactor (TACI), and B cell maturation antigen (BCMA), the expression of which may vary depending on the context (55).

Several recombinant antibodies and fusion proteins targeting components of the BAFF/APRIL system have been developed, however, none have progressed past phase-II trials (57). Of particular interest is atacicept (TACI-Ig), a fusion protein comprised of the extracellular domain of the naturally occurring TACI receptor and the Fc domain of human IgG. Atacicept binds the cytokines BAFF and APRIL, thereby preventing their interaction with surface receptors on B cells. Two phase-II clinical trials with atacicept – the ATAMS study in 255 patients with relapsing MS (58) and the ATON study in 34 patients (initially planned for 80) with unilateral optic neuritis (59) have been prematurely terminated due to increased disease activity in the atacicept treatment group in ATAMS and twice as many atacicept-treated patients converting to clinically definite MS in ATON, compared with placebo. Relapse-rates normalized to those of placebo-treated patients, and B cells and immunoglobulin recovered to reference levels after atacicept cessation (58). The differential effects of anti-CD20 mAbs and atacicept in MS may be explained by the fact that anti-CD20 mAbs have a broader depleting pattern, while atacicept has a significant impact on Breg without sufficiently depleting pathogenic B cell sub-sets, or it reduces serum immunoglobulins and disrupts non-specific Fc receptor blockade, which could have a therapeutic benefit. In addition, receptors for BAFF and APRIL also expressed on some T cells and regulatory cells were found to have more pleiotropic roles, which may include protective pathways that may be disrupted by their blockade. There is also evidence that suggests that APRIL is a negative regulator of autoimmunity and that atacicept preferentially targets naive B cells, plasmablasts, and plasma cells but has a lesser effect on memory-B cells, which are the relevant disease-promoting subset, resulting in a relative increase in memory-B cells after depletion of soluble BAFF and APRIL (57,60). Overall, the experience with atacicept suggests that the roles of B cells and humoral immunity in MS are complex and not fully understood, therefore calling for caution when testing new agents.

### Inhibition of Bruton's tyrosine kinase

Bruton’s tyrosine kinase (BTK) is a key cytoplasmic enzyme that mediates B cell signaling via a variety of cell surface molecules, including BCR, resulting in multiple downstream immune effects (61). Administration of BTK inhibitors leads to B cell inhibition, which is rapidly reversible upon treatment cessation, and to suppression of EAE disease activity (62). Evobrutinib is a highly specific, irreversible, oral BTK inhibitor, which was also shown to inhibit M1 macrophage and cytokine release, and promote M2 polarization of human monocytes *in vitro* (63). In a recently-completed phase-II trial, patients with RRMS or SPMS treated with evobrutinib showed reduced number of Gd + lesions on MRI scans and a clinically-relevant trend toward a reduction in ARR (64). Treatment was well-tolerated, and the main adverse events were asymptomatic, reversible transaminase and lipase elevations (64). The dual mechanism of action of evobrutinib, which targets pathogenic adaptive and innate immunity, and its favorable benefit-risk profile, support its further clinical development.

### The effects of other approved MS therapies on B cells

The complex, multi-player immune pathogenesis of MS, which provides multiple sites for therapeutic intervention on one hand, and the various mechanisms by which B cells contribute to the pathogenesis of MS along with the success of anti-CD20 therapies in MS, on the other hand, propelled studies on the effects of other MS drugs on B cells. Indeed, essentially all other approved therapies for MS, some of which have been designed to target T cells, were found to have some effects on the frequency, phenotype, trafficking, function, or responses of B cells, which may contribute to their therapeutic activity (11,32,57,65-67) (Table 2).

## FUTURE DIRECTIONS

Despite the therapeutic success of B cell depletion in MS, several important questions and challenges still exist. It is not completely understood why some patients do not respond adequately to B cell depletion therapies, and which MS patients will benefit best from B cell-directed therapies. While anti-CD20 mAbs deplete mainly circulating B cells, it is unclear whether B cells should be depleted also from the CNS or other compartments (eg, bone marrow or lymphatic tissue). The long-term safety of prolonged B cell depletion and the duration of depletion of peripheral B cells are still unknown. Maintenance therapies that would prevent re-emergence of pathogenic B cells after cessation of anti-B cell therapies or divert them toward a regulatory profile should be developed. Some researchers believe that there is no need for using more than one anti-CD20 mAb in MS; however, the selection between several anti-CD20 mAbs with different ADCC or CDC activities (eg, for patients who may respond better to CDC-mediated depletion because of polymorphisms in the Fc receptor regions that may reduce the binding of the depleting Ab to its receptor on effector immune cells and decrease ADCC), routes, or speed of administration may be useful in personalizing treatment for a wider range of patients exhibiting different needs and disease characteristics. Using mAbs to CD19, such as inebilizumab (MEDI-551), which targets also pro-B cells, plasmablasts, and plasma cells may provide more complete and prolonged B cell depletion (68). However, it is still unclear whether depleting broader range of B cells entails greater clinical benefits or more potentially serious adverse events, which result from negatively affecting B cell reconstitution due to the elimination of earlier stages in the bone marrow or reducing humoral immunity by elimination of antibody-producing cells. Additional approaches with a potential to target B cells that have not yet been explored as MS treatments or have not progressed past phase-II clinical trials include the use of other B cell-targeting mAbs such as epratuzumab (anti-CD22, a negative regulator of BCR-derived activation signals), daratumumab (anti-CD38 that depletes plasmablasts and some plasma cells), LTbR-IgG (anti-lymphotoxin beta receptor that would reduce the formation of ectopic germinal centers), NNC114-0005 (anti-IL21, an important cytokine for Ab formation), otilimab (anti-GM-CSF that blocks pro-inflammatory myeloid cell response), belimumab and talabumab (anti-BAFF), VAY736 (anti-BAFF receptor), hBCMA-Fc (human BCMA fused to IgG1 Fc), and mAbs to co-stimulatory molecules that would prevent B cell activation (32,57). In addition, several small molecules that target B cell signaling (through BTK, PI3 kinase, or Janus kinases), proteasome that is involved with plasma cell differentiation, or Epstein-Barr virus, which infects B cells and is believed to be involved in MS etiology, may provide novel mechanisms of targeting B cells and possibly other cells involved in the immune pathogenesis of MS (67).

Despite recent major advances toward a better understanding of the role of B cells in MS, there is still much left to be explored and discovered. Development of more effective and safer therapies directed at B cells should focus on compounds that also target specific plasma cells or do not affect Breg, and depends on enhanced understanding and further research into B cell biology, as well as a better understanding of MS pathogenesis.
